# A Orientina Alivia o Estresse Oxidativo e a Apoptose na Cardiomiopatia Diabética por Meio do Eixo Lncrna H19/Mir-103-3p/ALDH2/PI3K/AKT

**DOI:** 10.36660/abc.20240885

**Published:** 2025-07-29

**Authors:** Xun Wang, Xiaofang Xiong, Wei Jiang, Shanshan Xu, Jun Li

**Affiliations:** 1 Wuhan Third Hospital Wuhan China Wuhan Third Hospital, Wuhan - China

**Keywords:** Cardiomiopatias, Apoptose, Estresse Oxidativo

## Abstract

**Fundamento:**

A cardiomiopatia diabética (CMD) é uma complicação cardiovascular irreversível do diabetes mellitus, caracterizada por remodelação cardíaca prejudicial. A orientina, um flavonoide hidrossolúvel presente em muitas plantas medicinais, exerce diversos efeitos farmacológicos.

**Objetivos:**

Investigar os efeitos cardioprotetores da orientina em condições diabéticas e elucidar os mecanismos associados aos RNAs não codificantes.

**Métodos:**

O modelo CMD induzido por estreptozotocina foi estabelecido pelo uso combinado de estreptozotocina e uma dieta rica em gordura. A estrutura e a função cardíaca em camundongos com diabetes mellitus foram avaliadas por meio de análises histológicas e ecocardiográficas. Masson, TUNEL, western blot e ELISA em corações de camundongos foram realizados para analisar fibrose cardíaca, apoptose e estresse oxidativo. Os níveis de expressão de lncRNA H19, miR-103-3p, ALDH2 e proteínas relacionadas a PI3K/AKT em células cardíacas de camundongos e HL-1 foram avaliados por qPCR em tempo real ou western blot. O nível de significância foi estabelecido em p<0,05.

**Resultados:**

A orientina melhorou a função cardíaca e amenizou a lesão cardíaca em camundongos diabéticos. A orientina inibiu a fibrose cardíaca, reduziu a apoptose dos cardiomiócitos e aumentou a atividade de enzimas antioxidantes. Em condições patológicas, os níveis de H19 e ALDH2 foram reduzidos, enquanto os níveis de miR-103-3p aumentaram, o que foi revertido pela orientina. A H19 aumentou a expressão de ALDH2 ligando-se a miR-103-3p e ativou a via PI3K/AKT em células HL-1 tratadas com altos níveis de glicose. A depleção de H19 ou o inibidor de PI3K reverteram os efeitos da orientina na apoptose e no estresse oxidativo em células HL-1 sob condições de altos níveis de glicose.

**Conclusões:**

Esses achados revelam um mecanismo protetor da orientina na CMD, que envolve a regulação do eixo de sinalização H19/miR-103-3p/ALDH2/PI3K/AKT, fornecendo uma estratégia potencial para o tratamento da CMD.

## Graphical abstract

**Figura Central:**
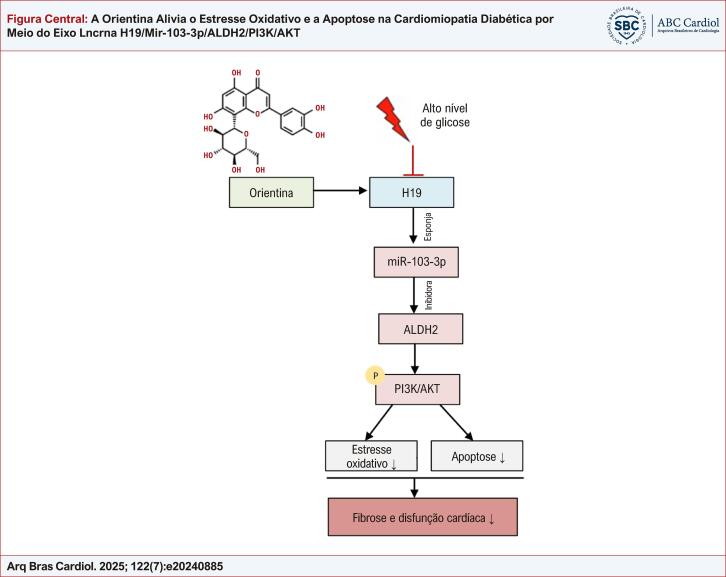
A Orientina Alivia o Estresse Oxidativo e a Apoptose na Cardiomiopatia Diabética por Meio do Eixo Lncrna H19/Mir-103-3p/ALDH2/PI3K/AKT

## Introdução

A diabetes mellitus (DM) é uma doença metabólica cuja prevalência continua a aumentar e é caracterizada por hiperglicemia resultante da secreção inadequada de insulina, resistência à insulina ou deficiência de glucagon. Hipersecreção.^1^ A cardiomiopatia diabética (CMD) é uma manifestação cardíaca do DM caracterizada por disfunção diastólica do ventrículo esquerdo e hipertrofia nos estágios iniciais,mais tarde por insuficiência cardíaca com função sistólica diminuída.^2^ Está bem estabelecido que as alterações estruturais e funcionais cardíacas em condições de CMD estão intimamente associadas ao estresse oxidativo persistente induzido por hiperglicemia, resultando emcardiomiócitomorte e disfunção cardíaca.^3-5^ Além disso, quando o coração está gravemente danificado, o excesso de colágeno leva à fibrose e disfunção cardíaca^6^ Até o momento, os mecanismos específicos associados à CMD são amplamente desconhecidos e não há tratamento eficaz para a doença. Portanto, é necessário identificar novos agentes ou alvos terapêuticos.

Os lncRNAs (long non-coding RNAs) desempenham funções regulatórias importantes em diversos processos e doenças celulares.^7^ Os microRNAs (miRNAs) participam das funções regulatórias de diversas atividades fisiológicas.^8^ Estudos sugerem que expressões anormais de ncRNAs estão envolvidas na progressão de doenças humanas, incluindo a CMD.^9-11^ Evidências também indicam que o tratamento com medicamentos (como a metformina) para DM pode alterar a expressão de ncRNA,^12^ sugerindo que a modulação da expressão de ncRNA pode ser um mecanismo importante no tratamento do DM. O LncRNA H19 participa dos processos regulatórios do metabolismo da glicose, metástase tumoral, diferenciação muscular, etc.^13^ O H19 foi identificado como uma molécula crucial em doenças cardíacas, pois pode regular a CMD,^14^ a hipertrofia cardíaca^15^ e a fibrose.^16^ A aldeído desidrogenase 2 (ALDH2) desempenha um papel fundamental na função respiratória mitocondrial e na remodelação da função ventricular.^17,18^ Além disso, o ALDH2 alivia a lesão isquêmica na CMD por meio da ativação da via PI3K/AKT.^19^ Nossos experimentos preliminares mostraram que o H19 pode aumentar a expressão de ALDH2 em cardiomiócitos de camundongos. Utilizando ferramentas online, identificamos o miR-103-3p como uma molécula-alvo do H19 e do ALDH2. No entanto, as funções regulatórias do eixo H19/miR-103-3p/ALDH2/PI3K/AKT na CMD ainda não foram investigadas.

A orientina é um componente flavonoide presente em plantas naturais, incluindo folhas de bambu, samambaia ocotillo, hidraste e benta.^20^ A orientina exerce diversos efeitos farmacológicos, como cardioproteção, neuroproteção, antiviral, antioxidante, antienvelhecimento, anticancerígeno e anti-inflamatório.^21-24^ Fu et al. relataram que a orientina é protetora contra o miocárdio tratado com isquemia/reperfusão e atenua a apoptose ao suprimir a ativação da via de apoptose mitocondrial.^25^ Li et al. relataram que a orientina atenua a remodelação cardíaca e reduz o infarto do miocárdio em camundongos através da via eNOS/NO.^26^ A orientina pode proteger podócitos e células endoteliais contra alterações morfológicas mitocondriais induzidas por altos níveis de glicose, apoptose, inflamação e estresse oxidativo.^27,28^ Além disso, a orientina pode resistir ao estresse oxidativo para exercer benefícios neuroprotetores^29^ e conferir cardioproteção contra reperfusão,^30^ ativando a sinalização PI3K/AKT. No entanto, os efeitos da orientina na CMD são amplamente desconhecidos.

Neste estudo, desenvolvemos modelos de CMD in vivo e in vitro para elucidar o papel do eixo de sinalização H19/miR-103-3p/ALDH2/PI3K/AKT nas funções regulatórias da orientina na CMD. Este estudo pode fornecer insights sobre o modo molecular de ação da orientina na doença cardíaca.

## Materiais e métodos

### Animais e tratamentos

Camundongos machos C57BL/6 (18 a 20 g; Beijing Vital River Laboratory) foram alojados em condições padrão (umidade de 60%; temperatura de 25 ± 1 ºC; ciclo claro/escuro de 12 h). Todos os procedimentos experimentais foram aprovados pelo Comitê de Pesquisa e Uso Animal da Wuhan Myhalic Biotechnology Co., Ltd (HLK-20240516003-003; 18 de julho de 2024).

O modelo de CMD foi estabelecido com uma dieta rica em gordura (Research Diet D12492) por 28 dias, seguida de jejum noturno e injeção intraperitoneal de estreptozotocina (50 mg/kg × 5 dias; 60256ES60; Shanghai Yeasen Biotechnology) dissolvida em tampão citrato de sódio (pH 4,5). Após 7 dias, os animais com hiperglicemia (glicemia ≥ 16,7 mmol/L) foram considerados camundongos DM^31^ e continuaram a ser alimentados com uma dieta rica em gordura. Os camundongos controle foram injetados com tampão citrato de sódio (pH 4,5) e alimentados com uma dieta normal. Os camundongos foram divididos em 5 grupos (n=8 por grupo): controle (camundongos controle tratados com solução salina normal); DM (camundongos DM tratados com solução salina normal); DM + Orientina (10 mg/kg) (camundongos DM tratados com 10 mg/kg de Orientina, MBS5750979; MyBiosource, Inc.); DM + Orientina (20 mg/kg) (camundongos DM tratados com 20 mg/kg de Orientina); DM + Orientina (40 mg/kg) (camundongos DM tratados com 40 mg/kg de Orientina). Orientina foi administrado intraperitonealmente uma vez ao dia (dissolvido em DMSO; um volume de 50 μl). Todos os grupos experimentais foram tratados por 12 semanas.

### Ecocardiografia

Os camundongos foram anestesiados com isoflurano a 0,5% e seus tórax foram raspados. A ecocardiografia em modo M foi realizada para avaliar a função cardíaca dos camundongos utilizando um Sistema de Microimagem de Alta Resolução (VeVo 770; VisualSonics Inc.). Parâmetros ecocardiográficos, incluindo diâmetro diastólico do ventrículo esquerdo (DVDVE), diâmetro sistólico do ventrículo esquerdo (DSVE), fração de ejeção do ventrículo esquerdo (FEVE) e encurtamento fracionário do ventrículo esquerdo (FSVE), foram medidos. Todas as medidas foram calculadas a partir de 5 ciclos cardíacos contínuos.

### Índice cardíaco

Os camundongos foram pesados e eutanasiados por inalação de CO_2_. A cavidade torácica foi aberta e o coração foi perfundido com 0,1 M de KCl. Após o coração parar de bater, o sangue residual foi removido com solução salina. O coração foi excisado e lavado três vezes. O coração foi seco em papel de filtro e pesado.

### Histologia

O tecido cardíaco de camundongo foi fixado em formalina a 10% (Top0372; Beijing Biotopped Life Sciences), desidratado e incluído em parafina. O tecido foi cortado em secções de 5 μm, colocadas em lâminas de vidro e coradas com hematoxilina-eosina (H&E; RS3390; Beijing G-Clone Biotechnology) utilizando o método padrão. A coloração tricrômica de Masson (RS3960l; Beijing G-Clone Biotechnology) foi utilizada para estimar a deposição de colágeno e a fibrose no coração. As alterações patológicas foram observadas ao microscópio Olympus CKX41. Cinco campos por secção cardíaca foram analisados utilizando o programa Image-Pro Plus 6.0.

### Detecção de lactato desidrogenase (LDH), creatina quinase-MB e troponina (cTnI)

Amostras de sangue foram obtidas do canto interno dos olhos dos camundongos e centrifugadas a 3.000×g por 15 minutos a 4°C. Indicadores de lesão cardíaca, incluindo LDH, CK-MB e cTnI no soro, foram medidos utilizando o Kit ELISA para LDH em Camundongo (ZK-M4619; Shenzhen Ziker Biotechnology), o Kit ELISA para CK-MB (ZK-M4805; Shenzhen Ziker Biotechnology) e o Kit ELISA para cTnI (JYM0409Mo; Wuhan Jiyinmei Biotechnology), respectivamente, seguindo os protocolos do fabricante.

### Coloração TUNEL

Para detectar a morte celular cardíaca, o ensaio TUNEL foi realizado utilizando o Kit de Detecção de Apoptose TUNEL FITC (A111-01; Nanjing Vazyme Biotechnology), seguindo as instruções do fabricante. Os núcleos foram marcados com DAPI (40727ES10; Shanghai Yeasen Biotechnology). As imagens foram obtidas utilizando um microscópio confocal Leica SP8. Cinco campos por secção cardíaca foram analisados utilizando o Image-Pro Plus 6.0.

### Células e tratamentos

Cardiomiócitos de camundongo HL-1 (ml096513; Shanghai mlbio) foram incubados em meio DMEM contendo 10% de SFB e 100 U/ml de penicilina-estreptomicina sob 5% de CO2/95% de ar a 37°C. Quando as células atingiram 70%-80% de confluência, foram expostas a glicose normal (5,5 mmol/L) ou glicose alta (33 mmol/L) e/ou orientina (2,5-20 μM) por 24 h. Para explorar os mecanismos de proteção da orientina, as células foram tratadas com 10 μM de LY294002 (um inibidor de PI3K; BES250679D; Shanghai BIOSEN Biotechnology) 2 h antes do tratamento com orientina.

### Transfecção

Cardiomiócitos de camundongo HL-1 foram transfectados com o vetor de superexpressão de H19 pcDNA3.1/H19 (H19), miR-103-3p ou siRNA direcionado a ALDH2 (si-ALDH2) (todos da Shanghai GenePharm Biotechnology) usando Lipofectamine 2000 (168019; Thermo Fisher Scientific, Inc.) seguindo os protocolos do fabricante. Vetor vazio (vetor), NC mimic e si-NC atuaram como controles negativos. Alterações nas expressões de RNA e proteína foram detectadas por qPCR e western blot 48 h após a transfecção.

### Ensaio CCK-8

Cardiomiócitos HL-1 foram semeados em placas de 96 poços (0,8-1 × 103 células/poço) durante a noite e tratados com glicose alta (33 mmol/L) e/ou orientina (2,5-20 μM) por 24 h. Em seguida, 10 µL de CCK-8 (40203ES60; Shanghai Yeasen Biotechnology) na concentração de 5 mg/mL foram adicionados a cada poço. Após o cultivo das células por 3-4 h a 37 °C, a absorbância a 450 nm foi medida utilizando um leitor de microplacas inteligente SMR16.1 (Wuhan USCNK IT LIFE SCIENCE INC.).

### Detecção de glutationa (GSH), superóxido dismutase (SOD), 4-hidroxinonenal (4-HNE) e malondialdeído (MDA).

Após homogeneizar 0,1 g de tecido cardíaco congelado com 1 mL de água destilada, o homogeneizado de tecido foi centrifugado a 12.000 rpm por 15 minutos. As células HL-1 foram lavadas com PBS (pH 7,2) e homogeneizadas em PBS contendo 0,5 mM de butil-hidroxitolueno. O homogeneizado foi centrifugado a 3.000 x g por 15 minutos a 4 °C. Glutationa (GSH), superóxido dismutase (SOD), 4-hidroxinonenal (4-HNE), malondialdeído (MDA) e ROS no sobrenadante foram detectados utilizando o Kit ELISA para GSH em camundongos (BLL-yx2756; Shanghai Baililai Biotechnology), o Kit ELISA para SOD (BLL-yx3040; Shanghai Baililai Biotechnology), o Kit ELISA para 4-HNE (YS03024B; Shanghai Yaji Biotechnology), o Kit ELISA para MDA (BLL-yx3039; Shanghai Baililai Biotechnology) e o Kit de Ensaio para ROS (DCFH-DA; S0033; Shanghai Beyotime Biotechnology), respectivamente, seguindo os protocolos do fabricante. Os níveis de ROS foram detectados por citometria de fluxo.

### RT-qPCR

Os RNAs totais foram isolados do tecido cardíaco e de células HL-1 utilizando o Extrator de RNA Total (zk3074; Shenzhen Zike Biotechnology) e transcritos reversamente em cDNA utilizando o Kit de Síntese de cDNA HiFiScript (AD502A; Shanghai Proteinssci Biotechnology), seguindo os protocolos do fornecedor. A reação de PCR foi realizada com o Power SYBR Green PCR Master Mix (Applied Biosystems) em um Sistema de PCR Rápido em Tempo Real 7900HT (Applied Biosystems). A expressão gênica foi calculada utilizando o método do ciclo limiar, que foi normalizado para GAPDH ou U6. As sequências de primers utilizadas para RT-qPCR são mostradas na Tabela 1.

**Tabela 1:** – Sequências de primers usadas para RT-qPCR

Alvo(rato)	Sequências de primers (5´-3´)
H19	F: CATTCTAGGCTGGGGTCAA
H19	R: GCCCTTCTTTTCCATTCTCC
miR-103-3p	F: ACACTCCAGCTGGGAGCAGCATTGTAC
miR-103-3p	R: TGGTGTCGTGGAGTCG
ALDH2	F: GCTGTTGTACCGATTGGCGGAT
ALDH2	R: GCGGAGACATTTCAGGACCATG
GAPDH	F: TGCACCACCAACTGCTTAG
GAPDH	R: GGATGCAGGGATGATGTTC
U6	F: CTCGCTTCGGCAGCACA
U6	R: AACGCTTCACGAATTGCGT

### Western blot

O isolamento de proteínas totais e a análise de Western blot foram conduzidos conforme descrito por Liu et al.^32^ Os anticorpos primários incluíram Bax (ab32503; 1:1500; Abcam), caspase3 clivada (#AF7022; 1:1500; afinidade), Bcl-2 (ab182858; 1:2000; Abcam), ALDH2 (#DF6358; 1:1500; afinidade), p-PI3K (#AF3241; 1:1500; afinidade), PI3K (#AF6241; 1:1500; afinidade), p-AKT (#AF0016; 1:1500; afinidade), AKT (#AF6261; 1:1500; afinidade) e GAPDH (#AF7021; 1:15000; afinidade). As bandas de proteína foram capturadas por substrato de quimioluminescência aprimorada (ES-0006; Wuhan FineTest Biotechnology). A análise densitométrica foi realizada utilizando o Image-Pro Plus 6.0.

### Ensaio do repórter de luciferase

Fragmentos selvagens (Wt) ou mutantes (Mut) de H19 e ALDH2 3’UTR contendo os sítios de ligação previstos para mmu-miR-103-3p foram inseridos no vetor Promega pmirGLO para construir os plasmídeos, incluindo H19-Wt/Mut e ALDH2-Wt/Mut, respectivamente. Células HL-1 foram semeadas em placas de 24 poços e transfectadas com esses plasmídeos repórter de luciferase, juntamente com o mimetizador de miR-103-3p ou o mimetizador de NC, utilizando Lipofectamine 2000. Após 48 h, a atividade da luciferase foi determinada utilizando o Kit de Ensaio do Repórter de Luciferase Promega.

### Análise estatística

A normalidade da distribuição dos dados foi verificada pelo teste de Shapiro-Wilk (p>0,05 para todos os grupos). Todos os valores são apresentados como média ± desvio padrão. ANOVA unidirecional com teste de Tukey foi utilizada para determinar diferenças significativas entre os grupos. P < 0,05 foi considerado estatisticamente significativo. Todas as análises foram realizadas no software SPSS v.21.0. Cada experimento foi repetido três vezes. Para estudos com animais, o tamanho da amostra foi determinado pela análise de poder estatístico (visando 80% de poder para detectar uma mudança de 30% nos desfechos primários em α=0,05).

## Resultados

### Orientina melhora a lesão cardíaca em camundongos DM

**Figura 1:**
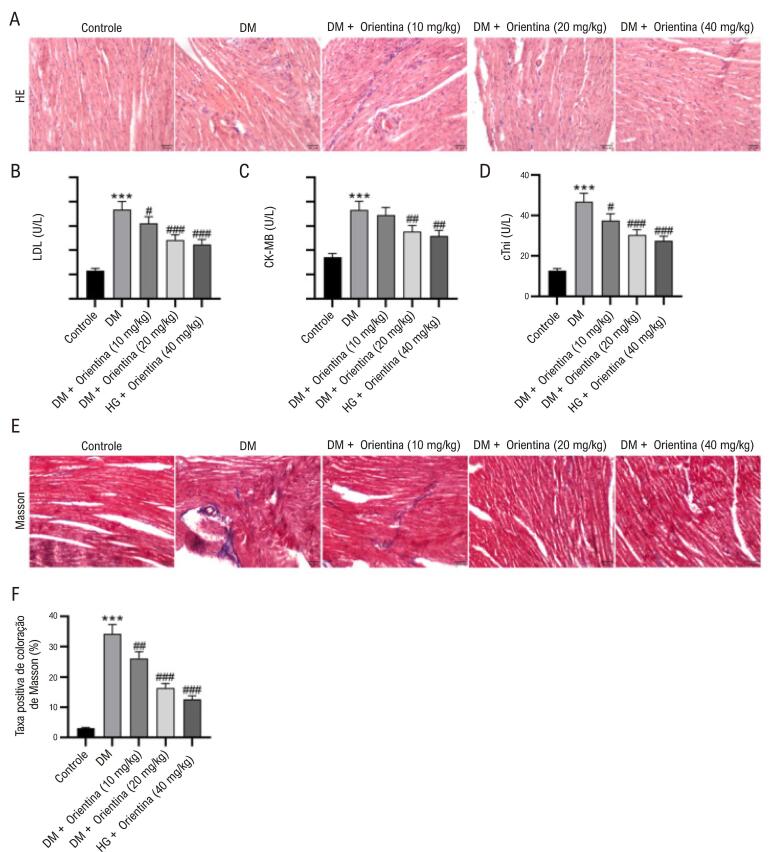
– Orientina melhora lesão cardíaca em camundongos com DM. (A) Alterações histológicas medidas pela coloração H&E. Barra de escala = 50 μm. (B-D) Efeitos da orientina nas atividades séricas de LDH, CK-MB e cTnI em camundongos com DM. ANOVA unidirecional. (E-F) Coloração de Masson de secção cardíaca e análise quantitativa. Barra de escala = 50 μm. ANOVA unidirecional. N = 8 por grupo. ***p < 0,01 vs grupo controle; #p < 0,05, ##p < 0,01, ###p < 0,001 vs grupo com DM. DM: diabetes mellitus.

Conforme mostrado na Tabela 2, comparado ao grupo controle, o grupo DM apresentou maiores níveis de glicose sanguínea, peso cardíaco e peso corporal (p<0,05). Orientina não teve impacto nos níveis de glicose sanguínea e peso corporal. Comparado ao grupo DM, orientina em doses de 20 e 40 mg/kg reduziu significativamente o peso cardíaco em camundongos (p<0,05). Dados de ecocardiografia em modo M na Tabela 3 indicaram que a FEVE e a FSVE foram reduzidas, e DVDVE e DSVE foram marcadamente aumentadas em camundongos diabéticos em comparação aos camundongos controle (p<0,05). Orientina em doses de 20 e 40 mg/kg elevou a FEVE e reduziu a DVDVE, e orientina em uma dose de 40 mg/kg elevou a FSVE e reduziu a DSVE (p<0,05). A coloração H&E da seção cardíaca no grupo controle exibiu morfologia normal de cardiomiócitos e fibras miocárdicas regulares. Entretanto, o tecido miocárdico no grupo com diabetes mellitus (DM) apresentou arquitetura desordenada evidente dos cardiomiócitos e hipertrofia miocárdica em comparação ao grupo controle. Orientina (20 e 40 mg/kg) melhorou a maioria das alterações patológicas (Figura 1A). As atividades séricas de LDH, CK-MB e cTnI, importantes indicadores de lesão cardíaca, foram estimadas. Os dados indicaram que as atividades séricas desses indicadores foram notavelmente aumentadas em camundongos diabéticos em comparação aos camundongos controle. Orientina nas doses de 20 e 40 mg/kg restaurou significativamente suas atividades em comparação aos camundongos diabéticos (Figura 1B-D). A coloração de Masson mostrou deposição significativa de colágeno no coração de camundongos com DM, que foi melhorada pela administração de Orientina (Figura 1E-F).

**Tabela 2:** – Efeitos da orientina sobre a glicemia, peso cardíaco, e peso corporal em camundongos diabéticos

Índices	Controle	DM	DM + Orientina (10 mg/kg)	DM + Orientina (20 mg/kg)	DM + Orientina (40 mg/kg)
Glicemia (mM)	8,24±1,14	22,31±1,78*	21,95±1,97	20,77±2,54	22,45±2,64
Peso do coração (mg)	111,25±9,14	138,67±11,09*	129,98±12,73	118,62±9,14#	120,08±8,98#
Peso corporal (g)	27,54±1,24	36,78±2,89*	35,22±1,79	36,02±3,11	37,15±2,52

Todos os valores são apresentados como média ± desvio padrão e analisados usando ANOVA unidirecional. N=8 por grupo. *p<0,05 vs grupo controle; #p<0,05 vs grupo DM. DM: diabetes mellitus.

**Tabela 3:** – Efeitos da orientina nas funções ventriculares esquerdas em camundongos diabéticos

Índices	Controle	DM	DM + Orientina (10 mg/kg)	DM + Orientina (20 mg/kg)	DM + Orientina (40 mg/kg)
%FEVE	610,91±3,25	38,57±2,37*	39,45±3,05	42,67±2,90#	53,74±3,75#
FSVE%	31,57±1,76	17,98±1,38*	19,64±1,12	19,99±1,24	25,86±1,47#
DVDVE (milímetros)	3,86±0,34	4,77±0,27*	40,61±0,66	4,06±0,86#	3,99±0,25#
DSVE (milímetros)	2,61±0,11	3,68±0,41*	3,44±0,66	3,38±0,14	2,76±0,09#

Todos os valores são apresentados como média ± desvio padrão e analisados usando ANOVA unidirecional. N=8 por grupo. *p<0,05 vs grupo controle; #p<0,05 vs grupo DM. DM: diabetes mellitus; DVDE: diâmetro diastólico do ventrículo esquerdo; DSVE: diâmetro sistólico do ventrículo esquerdo; FEVE: fração de ejeção do ventrículo esquerdo; FSVE: encurtamento fracionário do ventrículo esquerdo.

### Orientina alivia a apoptose miocárdica e o estresse oxidativo em camundongos com DM.

**Figura 2:**
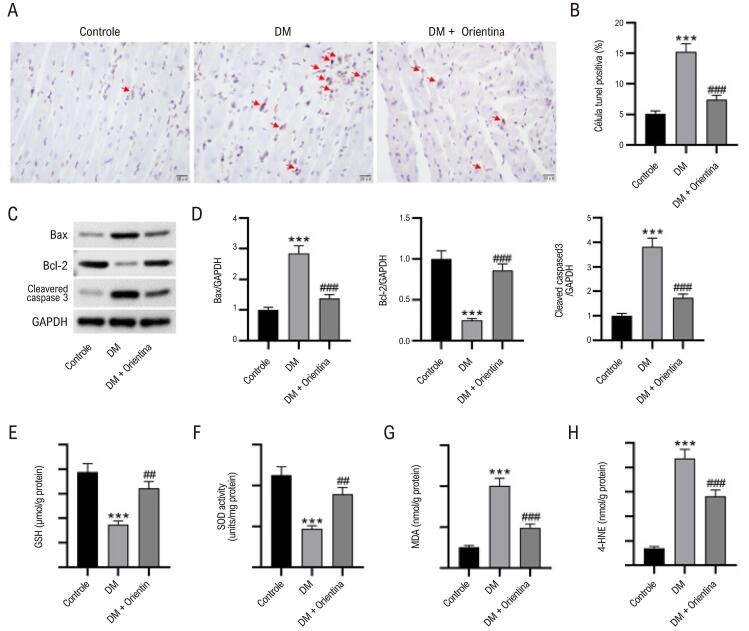
– Orientina alivia a apoptose miocárdica e o estresse oxidativo em camundongos com DM. (A-B) Coloração TUNEL da secção cardíaca e análise quantitativa. Barra de escala = 20 μm. ANOVA unidirecional. (C-D) Dados de Western blot mostrando os níveis de proteínas relacionadas à apoptose nos corações dos camundongos. ANOVA unidirecional. (E-H) Atividades de GSH e SOD, bem como níveis de MDA e 4-HNE nos corações dos camundongos. ANOVA unidirecional. N = 8 por grupo. ***p < 0,01 vs grupo controle; ##p < 0,01, ###p < 0,001 vs grupo com DM.

Como a orientina, na dose de 40 mg/kg, apresentou os melhores efeitos cardioprotetores, utilizamos essa dose em experimentos subsequentes. O grupo diabético apresentou um número maior de cardiomiócitos TUNEL-positivos no coração do que o grupo controle. A administração de orientina reverteu essa tendência e reduziu a apoptose dos cardiomiócitos (Figura 2A-B). A orientina também restaurou os níveis de proteínas relacionadas à apoptose em camundongos diabéticos, como demonstrado pela regulação positiva de Bcl-2 e regulação negativa de Bax e pela clivação de caspase-3 no grupo diabético, em comparação com o grupo diabético (Figura 2C-D). Avaliamos os níveis de parâmetros relacionados ao estresse oxidativo. Camundongos diabéticos apresentaram reduções significativas nas atividades cardíacas de GSH e SOD e aumentos nos níveis de MDA e 4-HNE em comparação com o grupo controle, que foram notavelmente restaurados pela orientina (Figura 2E-H).

### Efeitos da orientina no eixo H19/miR-103-3p/ALDH2/PI3K/AKT em camundongos DM

**Figura 3:**
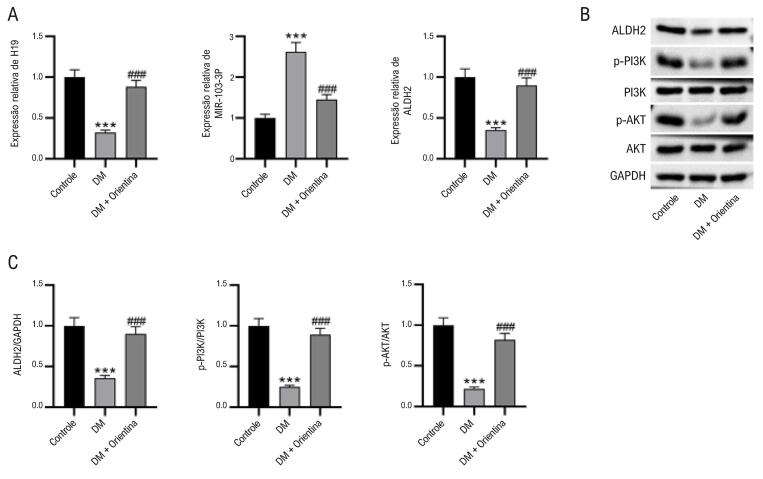
– Efeitos da orientina no eixo H19/miR-103-3p/ALDH2/PI3K/AKT em camundongos com DM. (A) Dados de RT-qPCR mostrando as expressões de H19, miR-103-3p e ALDH2 em corações de camundongos. ANOVA unidirecional. (B-C) Western blot mostrando os níveis de ALDH2 e PI3K e AKT fosforilados em corações de camundongos. ANOVA unidirecional. N = 8 por grupo. ***p < 0,01 vs grupo controle; ###p < 0,001 vs grupo com DM.

Dados de RT-qPCR mostraram que, em camundongos diabéticos, H19 e ALDH2 no miocárdio foram notavelmente regulados negativamente, enquanto miR-103-3p foi regulado positivamente, em comparação com camundongos controle. Seus níveis de expressão foram restaurados após o tratamento com orientina (Figura 3A). Além disso, os níveis proteicos de ALDH2 e PI3K e AKT fosforilados foram marcadamente menores no grupo com diabetes do que no grupo controle, sendo revertidos no grupo tratado com orientina (Figura 3B-C).

### Orientina reduz o estresse oxidativo e a apoptose desencadeados por altos níveis de glicose em células HL-1.

**Figura 4:**
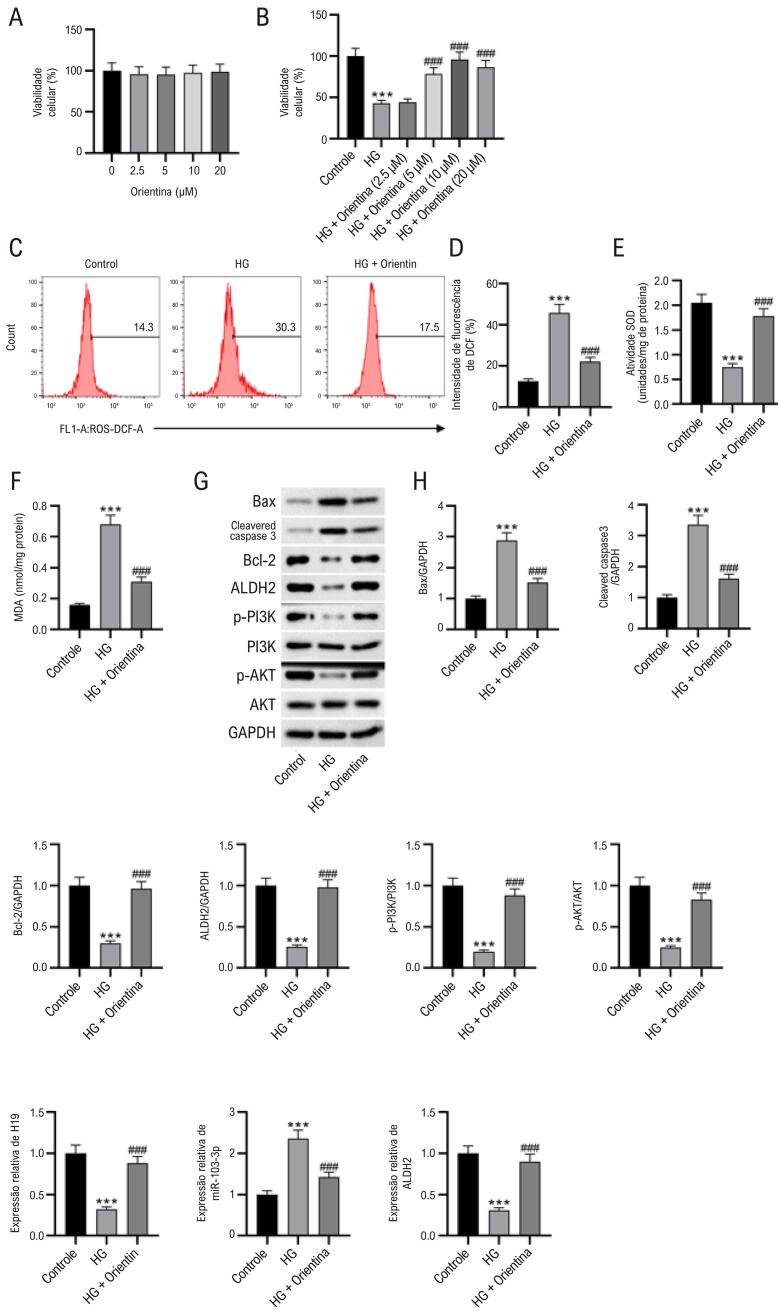
– Orientina reduz o estresse oxidativo e a apoptose desencadeados por altos níveis de glicose em células HL-1. (A-B) Viabilidade de células HL-1 após tratamento com orientina e/ou altos níveis de glicose, medida por CCK-8. ANOVA unidirecional. (C-F) Efeitos da orientina sobre os níveis de ROS e MDA e atividades de SOD em células HL-1. ANOVA unidirecional. (G-H) Western blot mostrando os níveis de Bax, Bcl-2, caspase-3 clivada, ALDH2 e PI3K e AKT fosforilados em cardiomiócitos HL-1. ANOVA unidirecional. (I) Dados de RT-qPCR mostrando as expressões de H19, miR-103-3p e ALDH2 em cardiomiócitos HL-1. ANOVA unidirecional. N = 3 por grupo. ***p < 0,01 vs grupo controle; ###p < 0,001 vs grupo DM.

Os resultados do CCK-8 indicaram que a orientina em doses de 2,5 a 20 μM não influenciou a viabilidade das células HL-1 (Figura 4A). A orientina em doses de 5, 10 e 20 μM resgatou a viabilidade das células HL suprimida por altos níveis de glicose (Figura 4B), e 10 μM de orientina apresentaram os efeitos mais significativos. Utilizamos 10 μM de orientina em experimentos subsequentes. Conforme apresentado nas Figuras 4C-F, altos níveis de glicose aumentaram os níveis de ROS e MDA e diminuíram as atividades de SOD, que foram revertidas pelo pré-tratamento com orientina. O pré-tratamento com orientina restaurou os níveis proteicos de Bax, Bcl-2, caspase-3 clivada, ALDH2 e PI3K e AKT fosforilados em cardiomiócitos HL-1 tratados com altos níveis de glicose (Figura 4G-H). Além disso, as expressões reduzidas de H19 e ALDH2 e o aumento da expressão de miR-103-3p em células HL-1 tratadas com alto teor de glicose foram revertidas pelo pré-tratamento com orientina (Figura 4I).

### O eixo H19/miR-103-3p/ALDH2 regula o estresse oxidativo e a apoptose em células HL-1

**Figura 5:**
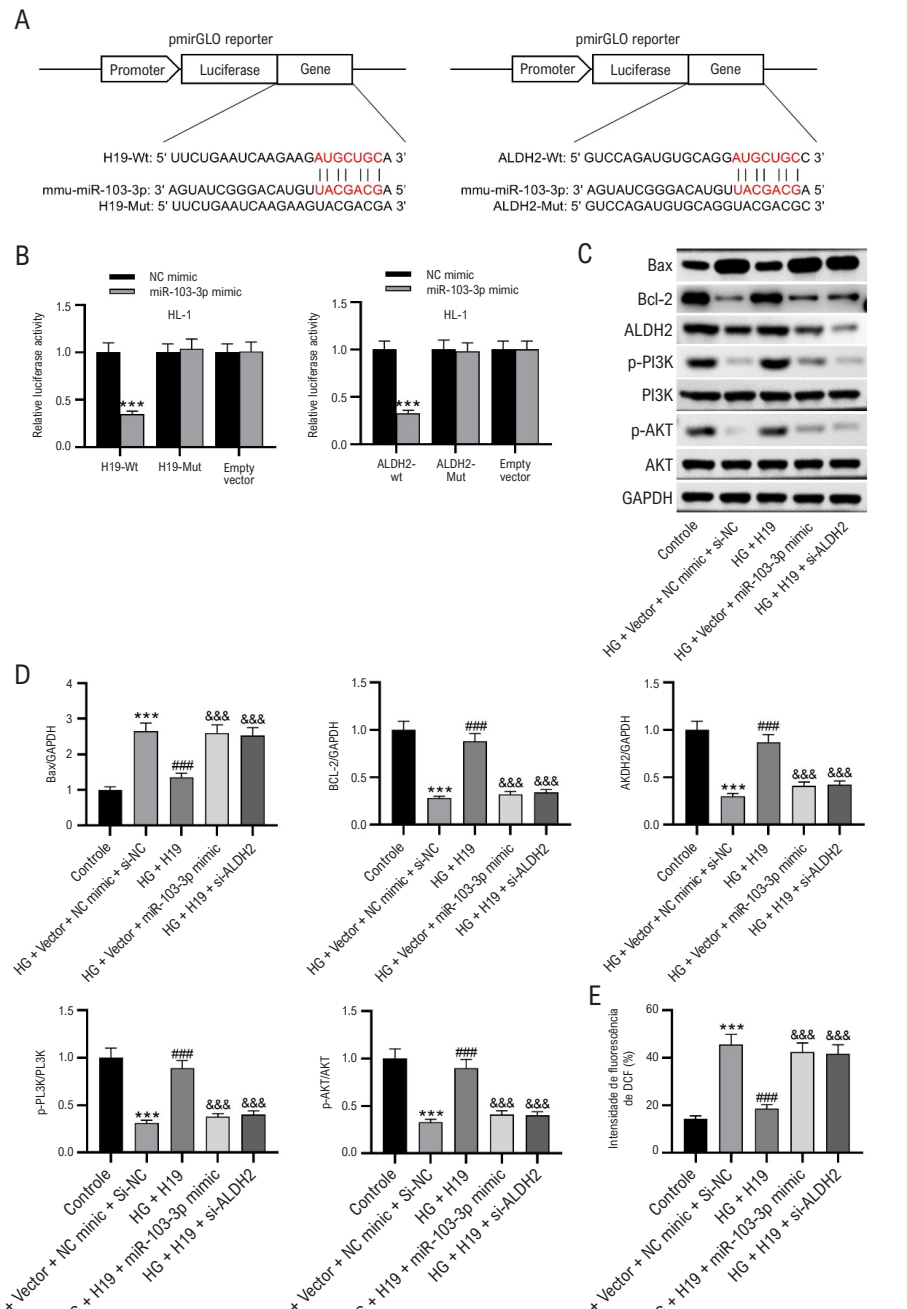
– O eixo H19/miR-103-3p/ALDH2 regula o estresse oxidativo e a apoptose em células HL-1. (A) Diagrama representando os sítios de ligação entre H19/ALDH2 e miR-103-3p. (B) Ensaio de repórter de luciferase mostrando atividades de luciferase após cotransfecções com H19-Wt/Mut ou ALDH1-Wt/Mut e mimetizador de miR-103-3p. ANOVA bidirecional. ***p<0,01 vs. grupo mimetizador NC. (C-D) Western blot mostrando níveis de Bax, Bcl-2, caspase3 clivada, ALDH2 e PI3K e AKT fosforilados em cardiomiócitos HL-1. ANOVA unidirecional. (E) Níveis de ROS em cardiomiócitos HL-1. ANOVA unidirecional. N=3 por grupo. ***p<0,01 vs. grupo controle; ###p<0,001 vs grupo HG + Vetor + NC mimic + si-NC; &&&p<0,001 vs grupo HG + H19.

Os sítios de ligação onde H19 ou ALDH2 interagem com miR-103-3p são mostrados na Figura 5A. A sequência H19/ALDH2 ou sua versão mutante foi cotransfetada em células HL-1 com o mimetizador miR-103-3p. Os dados indicaram reduções significativas nas atividades da luciferase após cotransfecções com H19-Wt ou ALDH1-Wt e mimetizador miR-103-3p, enquanto as cotransfecções com H19-Mut ou ALDH1-Mut e mimetizador miR-103-3p não alteraram as atividades da luciferase (Figura 5B). Descobrimos que a superexpressão de H19 restaurou os níveis proteicos de Bax, Bcl-2, caspase-3 clivada, ALDH2 e PI3K e AKT fosforilados em cardiomiócitos HL-1 tratados com altos níveis de glicose, que foram revertidos pela superexpressão de miR-103-3p ou pela depleção de ALDH2 (Figura 5C-D). Além disso, a superexpressão de H19 inibiu a produção de ROS desencadeada por altos níveis de glicose em células HL-1, mas a superexpressão de miR-103-3p ou a depleção de ALDH2 anularam os efeitos da superexpressão de H19 (Figura 5E).

### A depleção de H19 ou inibidor de PI3K reverte os efeitos da orientina em células HL-1

**Figura 6:**
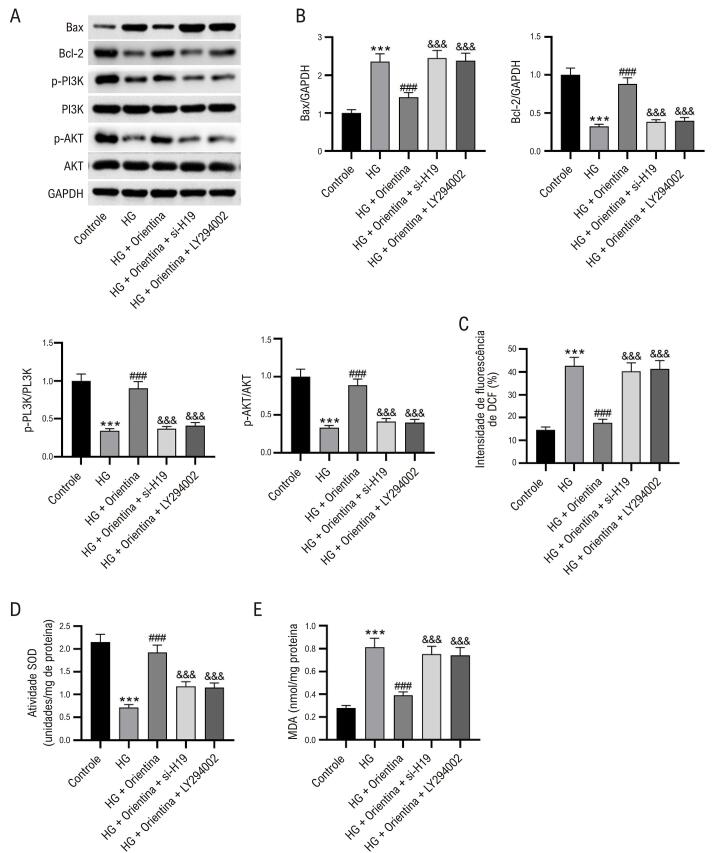
– A depleção de H19 ou o inibidor de PI3K revertem os efeitos da orientina em células HL-1. (A-B) Western blot mostrando os níveis de Bax, Bcl-2, caspase-3 clivada, ALDH2 e PI3K e AKT fosforilados em cardiomiócitos HL-1. ANOVA unidirecional. (C-E) Níveis de ROS e MDA e atividades de SOD em células HL-1. ANOVA unidirecional. N = 3 por grupo. ***p < 0,01 vs grupo controle; ###p < 0,001 vs grupo HG; &&&p < 0,001 vs grupo HG + Orientina.

Para os experimentos de resgate, cardiomiócitos HL-1 sob condições de alta glicose foram tratados com orientina e/ou com depleção de H19 ou inibidor de PI3K (LY294002). Os resultados mostraram que as alterações nos níveis de Bax, Bcl-2, caspase-3 clivada, ALDH2 e PI3K e AKT fosforiladas causadas pelo tratamento com orientina foram notavelmente revertidas pela redução de H19 ou pelo tratamento com LY294002 (Figura 6A-B). Além disso, a redução de H19 ou o tratamento com LY294002 neutralizaram os efeitos da orientina sobre os níveis de ROS e MDA e sobre as atividades da SOD em células HL-1 tratadas com alta glicose (Figura 6C-E).

## Discussão

Neste estudo, o tratamento com orientina aliviou a deterioração da função cardíaca em camundongos com CMD e protegeu contra fibrose cardíaca, apoptose e estresse oxidativo induzidos por diabetes. Mecanicamente, a orientina restaurou as alterações nos níveis de H19, miR-103-3p e ALDH2, bem como na via PI3K/AKT, causadas pela hiperglicemia. Esta investigação, pela primeira vez, revela que o eixo H19/miR-103-3p/ALDH2/PI3K/AKT desempenha um papel fundamental na ação cardioprotetora da orientina na CMD.

A orientina foi proposta como um composto promissor para o tratamento de doenças cardiovasculares. A orientina pode exercer efeitos antioxidantes e antiapoptóticos em células endoteliais vasculares tratadas com LDL-ox e pode proteger contra a aterosclerose.^33^ A orientina diminui a resposta inflamatória, a fibrose e a apoptose de cardiomiócitos em camundongos após infarto do miocárdio. Além disso, a orientina atenua a apoptose e o estresse oxidativo em cardiomiócitos expostos à hipóxia.^25,26^ Complicações macrovasculares do diabetes, incluindo CMD, ocorrem por meio de múltiplos mecanismos induzidos por hiperglicemia nos quais o estresse oxidativo tem um papel central.^34^ O estresse oxidativo cardíaco está associado à redução do desempenho e da contratilidade cardíacos e ao aumento da fibrose e hipertrofia cardíacas, resultando em disfunção cardíaca grave e eventos cardíacos fatais.^35^ O estresse oxidativo mediado por hiperglicemia persistente pode ativar a sinalização apoptótica no coração para induzir a morte de cardiomiócitos.^36,37^ Neste estudo, a orientina não influenciou os níveis de glicose sanguínea em camundongos DM. Ainda assim, ela melhorou significativamente a disfunção cardíaca induzida por diabetes em camundongos, independentemente da modulação dos níveis de glicose sanguínea, possivelmente por meio da inibição do estresse oxidativo cardíaco e da apoptose. A ecocardiografia em modo M confirmou que a orientina melhorou a disfunção cardíaca induzida por hiperglicemia. Análises morfológicas e de coloração demonstraram ainda que a orientina melhorou o dano ao tecido cardíaco induzido por diabetes. LDH, CK-MB e cTnI são marcadores bioquímicos importantes que refletem o grau de lesão cardíaca.^38^ Neste caso, a orientina reduziu acentuadamente os níveis de LDH, CK-MB e cTnI no soro de camundongos com DM.

A rede lncRNA-miRNA-mRNA ceRNA tem sido relatada como envolvida na regulação dos processos patológicos da CMD.^39-41^ H19, um lncRNA abundante no sistema cardiovascular, atua como um ceRNA para desempenhar um papel em complicações relacionadas ao diabetes.^42,43^ Além disso, H19 está emergindo como um regulador potencialmente importante da doença cardíaca.^44^ Conforme relatado, a superexpressão de H19 melhora a disfunção ventricular esquerda no DM, reduzindo a apoptose e a fibrose dos cardiomiócitos.^14^ A decocção de Shengjie Tongyu protege contra lesão cardíaca diabética por meio da regulação positiva de H19.^45^ Embora tenha sido relatado que a orientina confere cardioproteção no nível proteico, não foi investigado se a orientina poderia modular a rede de ceRNA. Aqui, confirmamos que a orientina restaurou a expressão de H19 e diminuiu a expressão de miR-103-3p *in vivo* e *in vitro*. H19 poderia se ligar ao miR-103-3p como uma esponja. O miR-103-3p pertence à família miR-103/107 e foi relatado como aumentando a necrose programada dos cardiomiócitos em lesões isquêmicas do miocárdio.^46^

Além disso, o miR-103-3p promove hipertrofia e produção de ROS no modelo de insuficiência cardíaca.^47^ Os efeitos do miR-103-3p na cardiomiopatia dilatada (CMD) são desconhecidos. Aqui, demonstramos que o miR-103-3p anulou os efeitos inibitórios da superexpressão de H19 na apoptose e no estresse oxidativo em células HL-1 sob condições de alta glicose. Além disso, o H19 pode regular positivamente a ALDH2, um alvo do miR-103-3p, em células HL-1 tratadas com alta glicose. Yu et al. demonstraram que a ALDH2 é um mediador crucial na cardioproteção do pós-condicionamento isquêmico remoto por meio da via dependente de PI3K/AKT.^48^ A ALDH2 alivia a lesão isquêmica na CMD por meio da ativação da via PI3K/AKT.^19^ Descobrimos que os efeitos da H19 nos níveis de proteínas relacionadas à apoptose e à ALDH2/PI3K/AKT em condições de alta glicose foram revertidos pela superexpressão de miR-103-3p ou pela redução de ALDH2, sugerindo que a H19 pode ativar a via ALDH2/PI3K/AKT ao competir com miR-103-3p. É importante ressaltar que a orientina pode aumentar a expressão de ALDH2 em cardiomiócitos com alta glicose. Os efeitos da orientina na apoptose e no estresse oxidativo dos cardiomiócitos foram revertidos pelo siRNA H19 ou por um inibidor de PI3K, demonstrando o papel fundamental do eixo H19/miR-103-3p/ALDH2/PI3K/AKT na ação cardioprotetora da orientina. Apesar desses achados, diversas limitações merecem consideração. Primeiramente, nosso modelo in vivo empregou camundongos diabéticos induzidos por estreptozotocina alimentados com uma dieta rica em gordura. Embora isso replique características-chave da CMD humana, não captura totalmente a complexidade da fisiopatologia do diabetes tipo 2, incluindo a disfunção gradual das células β. Em segundo lugar, o estudo se concentrou no eixo H19/miR-103-3p/ALDH2/PI3K/AKT como a principal via mecanística; outras cascatas de sinalização ou RNAs não codificantes podem contribuir para os efeitos cardioprotetores da orientina. Em terceiro lugar, a segurança e a farmacocinética a longo prazo da orientina em modelos diabéticos permanecem inexploradas. Estudos futuros usando modelos genéticos de diabetes, abordagens multiômicas e avaliações de toxicidade crônica fortaleceriam a tradução clínica.

## Conclusão

Em conclusão, nosso estudo demonstra que a orientina melhora a CMD atenuando o estresse oxidativo e a apoptose através do eixo de sinalização H19/miR-103-3p/ALDH2/PI3K/AKT. Este trabalho não apenas revela um novo mecanismo molecular para os efeitos cardioprotetores da orientina, como também identifica H19 e ALDH2 como potenciais alvos terapêuticos para a CMD. Dada a origem natural da orientina, esses achados corroboram sua promessa translacional como estratégia complementar contra complicações cardíacas induzidas pelo diabetes.
