# Supplementary material for: A Orientina Alivia o Estresse Oxidativo e a Apoptose na Cardiomiopatia Diabética por Meio do Eixo Lncrna H19/Mir-103-3p/ALDH2/PI3K/AKT
Source: Arq Bras Cardiol. 2025 Jul 29;122(7):e20240885. [Article in Portuguese] doi: 10.36660/abc.20240885 (PMC12695092; doi:10.36660/abc.20240885)
Supplement: Translation

# Translation: Orientin Alleviates Oxidative Stress And Apoptosis In Diabetic Cardiomyopathy Via The Lncrna H19/Mir-103-3p/ALDH2/PI3K/AKT Axis

**DOI:** 10.36660/abc.20240885i

**Authors:** Xun Wang, Xiaofang Xiong, Wei Jiang, Shanshan Xu, Jun Li

**Affiliations:** 1 China Wuhan Third Hospital, Wuhan - China

**Keywords:** Cardiomyopathies, Apoptosis, Oxidative Stress

## Abstract

**Background:**

Diabetic cardiomyopathy (DCM) is an irreversible cardiovascular complication of diabetes mellitus characterized by detrimental cardiac remodeling. Orientin, a water-soluble flavonoid present in many medicinal plants, exerts various pharmacological effects.

**Objectives:**

To investigate the cardioprotective effects of orientin in diabetic conditions and elucidate the mechanisms associated with non-coding RNAs.

**Methods:**

The streptozotocin-induced DCM model was established by a combined use of streptozotocin and a high-fat diet. Cardiac structure and function in diabetes mellitus mice were evaluated using histological and echocardiographic analyses. Masson, TUNEL, western blot, and ELISA in mouse hearts were performed to analyze cardiac fibrosis, apoptosis, and oxidative stress. Expression levels of lncRNA H19, miR-103-3p, ALDH2, and PI3K/AKT-related proteins in mouse heart and HL-1 cells were evaluated using real-time qPCR or western blot. The significance level was set at p<0.05.

**Results:**

Orientin improved cardiac function and ameliorated cardiac injury in diabetic mice. Orientin inhibited cardiac fibrosis, reduced cardiomyocyte apoptosis, and increased the activities of antioxidant enzymes. In pathological conditions, H19 and ALDH2 levels were reduced while miR-103-3p levels increased, which were reversed by orientin. H19 upregulated ALDH2 expression by binding to miR-103-3p and activated the PI3K/AKT pathway in high glucose-treated HL-1 cells. H19 depletion or PI3K inhibitor reversed the effects of orientin on apoptosis and oxidative stress in HL-1 cells under high glucose conditions.

**Conclusions:**

These findings reveal a protective mechanism of orientin in DCM, which involves the regulation of the H19/miR-103-3p/ALDH2/PI3K/AKT signaling axis, providing a potential strategy for treating DCM.

## Introduction

Diabetes mellitus (DM) is a metabolic illness that continues to rise in prevalence and is characterized by hyperglycemia resulting from inadequate insulin secretion, insulin resistance, or glucagon hypersecretion.^1^ Diabetic cardiomyopathy (DCM) is a cardiac manifestation of DM characterized by left ventricular diastolic dysfunction and hypertrophy in the early stages, later by heart failure with decreased systolic function.^2^ It is well established that cardiac structural and functional changes under DCM conditions are closely associated with hyperglycemia-induced persistent oxidative stress, resulting in cardiomyocyte death and cardiac dysfunction.^3-5^ Additionally, when the heart is severely damaged, excessive collagen leads to cardiac fibrosis and dysfunction.^6^ To date, the specific mechanisms associated with DCM are largely unknown, and there is no effective treatment for DCM. Therefore, it is needed to identify new therapeutic agents or targets.

Long non-coding (lncRNAs) have important regulatory functions in numerous cellular processes and diseases.^7^ MicroRNAs (miRNAs) participate in the regulatory functions of a variety of physiological activities.^8^ Studies suggest that abnormal expressions of ncRNAs are involved in human disease progression, including DCM.^9-11^ Evidence also indicates that treatment with drugs (such as metformin) for DM can alter ncRNA expression,^12^ implying that the modulation of ncRNA expression might be an important mechanism in DM treatment. LncRNA H19 participates in the regulatory processes of glucose metabolism, tumor metastasis, muscle differentiation, etc.^13^ H19 has been identified as a crucial molecule in cardiac disease, as it can regulate DCM,^14^ cardiac hypertrophy,^15^ and fibrosis.^16^ Aldehyde dehydrogenase 2 (ALDH2) plays a key role in mitochondrial respiration function and ventricular function remodeling.^17,18^ Moreover, ALDH2 alleviates ischemic injury in DCM through activation of the PI3K/AKT pathway.^19^ Our preliminary experiments showed that H19 could upregulate ALDH2 expression in mouse cardiomyocytes. By using online tools, we identified miR-103-3p as a target molecule of H19 and ALDH2. However, the regulatory functions of the H19/miR-103-3p/ALDH2/PI3K/AKT axis in DCM have not been investigated.

Orientin is a flavonoid component present in natural plants, including bamboo leaves, ocotillo fern, goldenseal, and holy.^20^ Orientin exerts various pharmacological effects, such as cardioprotective, neuroprotective antiviral, antioxidant, antiaging, anticancer, and anti-inflammatory activities.^21-24^ Fu et al. reported that orientin is protective against ischemia/reperfusion-treated myocardium and attenuates apoptosis by suppressing the activation of the mitochondria apoptosis pathway.^25^ Li et al. reported that orientin mitigates cardiac remodeling and reduces myocardial infarction in mice through the eNOS/NO pathway.^26^ Orientin can protect podocytes and endothelial cells against high glucose-induced mitochondrial morphological changes, apoptosis, inflammation, and oxidative stress.^27,28^ Additionally, orientin can resist oxidative stress to exert neuroprotective benefits^29^ and confer cardioprotection against reperfusion^30^ by activating PI3K/AKT signaling. However, the effects of orientin on DCM are largely unknown.

In this study, we developed DCM models *in vivo* and *in vitro* to elucidate the role of the H19/miR-103-3p/ALDH2/PI3K/AKT signaling axis in the regulatory functions of orientin in DCM. This study might provide insights into the molecular mode of orientin’s action in heart disease.

## Materials and methods

### Animals and treatments

Male C57BL/6 mice (18 to 20 g; Beijing Vital River Laboratory) were housed under standard conditions (humidity of 60%; temperature at 25±1°C; 12-h light/dark cycle). All experimental procedures were approved by the Animal Research and Use Committee of Wuhan Myhalic Biotechnology Co., Ltd (HLK-20240516003-003; July 18, 2024).

The DCM model was established with a high-fat diet (Research Diet D12492) for 28 days, followed by fasting overnight and intraperitoneal injection with streptozotocin (50 mg/kg × 5 days; 60256ES60; Shanghai Yeasen Biotechnology) dissolved in sodium citrate buffer (pH 4.5). After 7 days, animals with hyperglycemia (blood glucose≥16.7 mmol/L) were considered DM mice^31^ and continued to be fed a high-fat diet. Control mice were injected with sodium citrate buffer (pH 4.5) and fed a normal diet. Mice were divided into 5 groups (n=8 per group): control (control mice treated with normal saline); DM (DM mice treated with normal saline); DM + Orientin (10 mg/kg) (DM mice treated with 10 mg/kg orientin, MBS5750979; MyBiosource, Inc.); DM + Orientin (20 mg/kg) (DM mice treated with 20 mg/kg orientin); DM + Orientin (40 mg/kg) (DM mice treated with 40 mg/kg orientin). Orientin was intraperitoneally administered once daily (dissolved in DMSO; a volume of 50 μl). All experimental groups were treated for 12 weeks.

### Echocardiography

Mice were anesthetized with 0.5% isoflurane, and their chests were shaved. M-mode echocardiography was performed to evaluate mouse cardiac function using a High-Resolution Micro-Imaging System (VeVo 770; VisualSonics Inc.). Echocardiography parameters, including left ventricular diastolic diameter (LVEDD), systolic diameter (LVEDS), ejection fraction (LVEF), and fractional shortening (LVFS) were measured. All measurements were averaged from 5 continuous cardiac cycles.

### Heart index

Mice were weighed and euthanized via CO_2_ inhalation. The chest cavity was opened, and the heart was perfused with 0.1 M KCl. After the heart stopped beating, the residual blood inside was removed with saline. Then, the heart was excised and washed three times. The heart was dried on filter paper and weighed.

### Histology

Mouse heart tissue was fixed in 10% formalin (Top0372; Beijing Biotopped Life Sciences), dehydrated, and embedded in paraffin. The tissue was cut into 5 μm sections, placed on glass slides, and stained with hematoxylin-eosin (H&E; RS3390; Beijing G-Clone Biotechnology) using the standard method. Masson’s trichrome staining (RS3960l; Beijing G-Clone Biotechnology) was used to estimate collagen deposition and fibrosis in the heart. Pathological changes were observed under an Olympus CKX41 microscope. Five fields per heart section were analyzed using Image-Pro Plus 6.0.

### Detection of lactate dehydrogenase (LDH), creatine kinase-MB, and troponin (cTnI)

Blood samples were obtained from the inner canthus of the mouse eyes and centrifuged at 3,000×g for 15 min at 4°C. Cardiac injury indicators, including LDH, CK-MB, and cTnI in serum, were measured using Mouse LDH ELISA Kit (ZK-M4619; Shenzhen Ziker Biotechnology), CK-MB ELISA Kit (ZK-M4805; Shenzhen Ziker Biotechnology), and cTnI ELISA Kit (JYM0409Mo; Wuhan Jiyinmei Biotechnology), respectively, following the manufacturer’s protocols.

### TUNEL staining

To detect cardiac cell death, the TUNEL assay was performed using the TUNEL FITC Apoptosis Detection Kit (A111-01; Nanjing Vazyme Biotechnology) following the manufacturer’s instructions. Nuclei were labeled with DAPI (40727ES10; Shanghai Yeasen Biotechnology). Images were obtained using a Leica SP8 confocal microscope. Five fields per heart section were analyzed using Image-Pro Plus 6.0.

### Cells and treatments

HL-1 mouse cardiomyocytes (ml096513; Shanghai mlbio) were incubated in DMEM containing 10% FBS and 100 U/ml penicillin-streptomycin under 5% CO_2_/95% air at 37°C. When cells reached 70%-80% confluence, they were exposed to normal glucose (5.5 mmol/L) or high glucose (33 mmol/L) or/and orientin (2.5-20 μM) for 24 h. To explore the protective mechanisms of orientin, cells were treated with 10 μM LY294002 (an inhibitor of PI3K; BES250679D; Shanghai BIOSEN Biotechnology) 2 h before orientin treatment.

### Transfection

HL-1 mouse cardiomyocytes were transfected with the H19 overexpression vector pcDNA3.1/H19 (H19), miR-103-3p, or siRNA targeting ALDH2 (si-ALDH2) (all from Shanghai GenePharm Biotechnology) using Lipofectamine 2000 (168019; Thermo Fisher Scientific, Inc.) following the manufacturer’s protocols. Empty vector (vector), NC mimic, and si-NC acted as negative controls. Changes in RNA and protein expressions were detected using qPCR and western blot 48 h after transfection.

### CCK-8 assay

HL-1 cardiomyocytes were seeded in 96-well plates (0.8-1×10^3^ cells/well) overnight and treated with high glucose (33 mmol/L) or/and orientin (2.5-20 μM) for 24 h. Then, 10 µL CCK-8 (40203ES60; Shanghai Yeasen Biotechnology) at a concentration of 5 mg/mL was added to each well. After cells were cultured for 3-4 h at 37°C, absorbance at 450 nm was measured using an SMR16.1 Smart Microplate Reader (Wuhan USCNK IT LIFE SCIENCE INC.).

### Detection of glutathione (GSH), superoxide Dismutase (SOD), 4-hydroxynonenal (4-HNE), and malondialdehyde (MDA)

After 0.1g frozen heart tissue was homogenized with 1 mL distilled water, the tissue homogenate was centrifuged at 12,000 rpm for 15 min. HL-1 cells were washed with PBS (pH 7.2) and homogenized in PBS containing 0.5 mM butylated hydroxytoluene. The homogenate was centrifuged at 3,000×g for 15 min at 4°C. Glutathione (GSH), superoxide Dismutase (SOD), 4-hydroxynonenal (4-HNE), malondialdehyde (MDA), and ROS in the supernatant were detected using Mouse GSH ELISA Kit (BLL-yx2756; Shanghai Baililai Biotechnology), SOD ELISA Kit (BLL-yx3040; Shanghai Baililai Biotechnology), 4-HNE ELISA Kit (YS03024B; Shanghai Yaji Biotechnology), MDA ELISA Kit (BLL-yx3039; Shanghai Baililai Biotechnology), and ROS Assay Kit (DCFH-DA; S0033; Shanghai Beyotime Biotechnology), respectively, following the manufacturer’s protocols. The ROS levels were detected by flow cytometry.

### RT-qPCR

Total RNAs were isolated from heart tissue and HL-1 cells using Total RNA Extractor (zk3074; Shenzhen Zike Biotechnology) and reversely transcribed into cDNA using HiFiScript cDNA Synthesis Kit (AD502A; Shanghai Proteinssci Biotechnology) following the supplier’s protocols. The PCR reaction was performed with Power SYBR Green PCR Master Mix (Applied Biosystems) on a 7900HT Fast Real-Time PCR System (Applied Biosystems). Gene expression was calculated using the threshold cycle method, which was normalized to GAPDH or U6. Primer sequences used for RT-qPCR are shown in Table 1.

**Table 1:** – Primer sequences used for RT-qPCR

Target (mouse)	Primer sequences (5´-3´)
H19	F: CATTCTAGGCTGGGGTCAAA
H19	R: GCCCTTCTTTTCCATTCTCC
miR-103-3p	F: ACACTCCAGCTGGGAGCAGCATTGTAC
miR-103-3p	R: TGGTGTCGTGGAGTCG
ALDH2	F: GCTGTTGTACCGATTGGCGGAT
ALDH2	R: GCGGAGACATTTCAGGACCATG
GAPDH	F: TGCACCACCAACTGCTTAG
GAPDH	R: GGATGCAGGGATGATGTTC
U6	F: CTCGCTTCGGCAGCACA
U6	R: AACGCTTCACGAATTTGCGT

### Western blot

Isolation of total proteins and western blot analysis were conducted as described by Liu et al. ^32^. Primary antibodies included Bax (ab32503; 1:1500; Abcam), cleaved caspase3 (#AF7022; 1:1500; affinity), Bcl-2 (ab182858; 1:2000; Abcam), ALDH2 (#DF6358; 1:1500; affinity), p-PI3K (#AF3241; 1:1500; affinity), PI3K (#AF6241; 1:1500; affinity), p-AKT (#AF0016; 1:1500; affinity), AKT (#AF6261; 1:1500; affinity), and GAPDH (#AF7021; 1:15000; affinity). Protein bands were captured by enhanced chemiluminescence substrate (ES-0006; Wuhan FineTest Biotechnology). Densitometry analysis was performed using Image-Pro Plus 6.0.

### Luciferase reporter assay

Wild type (Wt) or mutant (Mut) fragment of H19 and ALDH2 3’UTR containing the predicted binding sites of mmu-miR-103-3p was inserted into the Promega pmirGLO vector to construct the plasmids including H19-Wt/Mut and ALDH2-Wt/Mut, respectively. HL-1 cells were seeded into 24-well plates and transfected with these luciferase reporter plasmids together with miR-103-3p mimic or NC mimic using Lipofectamine 2000. At 48 h, luciferase activity was determined using the Promega Luciferase Reporter Assay Kit.

### Statistical analysis

The normality of data distribution was verified using the Shapiro-Wilk test (p>0.05 for all groups). All values are shown as means ± standard deviation. One/two-way ANOVA with a Tukey’s test was used to determine significant differences between groups. P<0.05 was considered statistically significant. All analyses were performed in SPSS v.21.0 software. Each experiment was repeated three times. For animal studies, the sample size was determined by statistical power analysis (targeting 80% power to detect a 30% change in primary endpoints at α=0.05).

## Results

### Orientin ameliorates heart injury in DM mice

As shown in Table 2, compared to the control group, the DM group exhibited higher blood glucose levels, heart weight, and body weight (p<0.05). Orientin had no impact on blood glucose levels and body weight. Compared to the DM group, orientin at doses of 20 and 40 mg/kg significantly reduced heart weight in mice (p<0.05). M-mode echocardiography data in Table 3 indicated that LVEF and LVFS were reduced, and LVEDD and LVEDS were markedly increased in diabetic mice compared to control mice (p<0.05). Orientin at doses of 20 and 40 mg/kg elevated LVEF and reduced LVEDD, and orientin at a dose of 40 mg/kg elevated LVFS and reduced LVEDS (p<0.05). H&E staining of the cardiac section in the control group exhibited normal morphology of cardiomyocytes and regular myocardial fibers. However, myocardial tissue in the DM group showed obvious disordered architecture of cardiomyocytes and myocardial hypertrophy compared to the control group. Orientin (20 and 40 mg/kg) ameliorated a majority of pathological changes (Figure 1A). Serum activities of LDH, CK-MB, and cTnI, important indicators of heart injury, were estimated. Data indicated that serum activities of these indicators were notably enhanced in diabetic mice compared to control mice. Orientin at doses of 20 and 40 mg/kg significantly restored their activities compared to diabetic mice (Figure 1B-D). Masson staining showed significant collagen deposition in the heart of DM mice, which was ameliorated by orientin administration (Figure 1E-F).

**Table 2:** – Effects of orientin on blood glucose, heart weight, and body weight in diabetic mice

Indexes	Control	DM	DM + Orientin (10 mg/kg)	DM + Orientin (20 mg/kg)	DM + Orientin (40 mg/kg)
Blood glucose (mM)	8.24±1.14	22.31±1.78*	21.95±1.97	20.77±2.54	22.45±2.64
Heart weight (mg)	111.25±9.14	138.67±11.09*	129.98±12.73	118.62±9.14#	120.08±8.98#
Body weight (g)	27.54±1.24	36.78±2.89*	35.22±1.79	36.02±3.11	37.15±2.52

All values are shown as means ± standard deviation and analyzed using one-way ANOVA. N=8 per group. *p<0.05 vs control group; #p<0.05 vs DM group. DM: diabetes mellitus

**Table 3:** – Effects of orientin on left ventricular functions in diabetic mice

Indexes	Control	DM	DM + Orientin (10 mg/kg)	DM + Orientin (20 mg/kg)	DM + Orientin (40 mg/kg)
LVEF%	61.91±3.25	38.57±2.37*	39.45±3.05	42.67±2.90#	53.74±3.75#
LVFS%	31.57±1.76	17.98±1.38*	19.64±1.12	19.99±1.24	25.86±1.47#
LVEDD (mm)	3.86±0.34	4.77±0.27*	4.61±0.66	4.06±0.86#	3.99±0.25#
LVEDS (mm)	2.61±0.11	3.68±0.41*	3.44±0.66	3.38±0.14	2.76±0.09#

All values are shown as means ± standard deviation and analyzed using one-way ANOVA. N=8 per group. *p<0.05 vs control group; #p<0.05 vs DM group. DM: diabetes mellitus; LVEDD: left ventricular diastolic diameter; LVESD: left ventricular systolic diameter; LVEF: left ventricular ejection fraction; LVSF: left ventricular fractional shortening.

**Figure 1:** – Orientin ameliorates heart injury in DM mice. (A) Histological changes measured by H&E staining. Scale bar=50 μm. (B-D) Effects of orientin on serum activities of LDH, CK-MB, and cTnI in DM mice. One-way ANOVA. (E-F) Masson staining of heart section and quantitative analysis. Scale bar=50 μm. One-way ANOVA. N=8 per group. ***p<0.01 vs control group; #p<0.05, ##p<0.01, ###p<0.001 vs DM group*.*

### Orientin alleviates myocardial apoptosis and oxidative stress in DM mice

Since 40 mg/kg, orientin showed the best cardioprotective effects, we used this dose in subsequent experiments. The DM group exhibited a higher number of TUNEL-positive cardiomyocytes in the heart than the control group. Orientin administration reversed this trend and reduced cardiomyocyte apoptosis (Figure 2A-B). Orientin also restored the levels of apoptosis-related proteins in diabetic mice, as demonstrated by upregulated Bcl-2 and downregulated Bax and cleaved caspase3 in the orientin group compared to the DM group (Figure 2C-D). We evaluated the levels of oxidative stress-related parameters. Diabetic mice exhibited significant reductions in heart GSH and SOD activities and increases in MDA and 4-HNE levels compared to the control group, which were notably restored by orientin (Figure 2E-H).

**Figure 2:** – Orientin alleviates myocardial apoptosis and oxidative stress in DM mice. (A-B) TUNEL staining of the heart section and quantitative analysis. Scale bar = 20 μm. One-way ANOVA. (C-D) Western blot data showing levels of apoptosis-related proteins in the mouse hearts. One-way ANOVA. (E-H) GSH and SOD activities, as well as MDA and 4-HNE levels in mouse hearts. One-way ANOVA. N=8 per group. ***p<0.01 vs control group; ##p<0.01, ###p<0.001 vs DM group.

### Effects of orientin on the H19/miR-103-3p/ALDH2/PI3K/AKT axis in DM mice

RT-qPCR data showed that, in diabetic mice, H19 and ALDH2 in the myocardium were notably downregulated, whereas miR-103-3p was upregulated, compared to control mice. Their expression levels were restored after orientin treatment (Figure 3A). Additionally, protein levels of ALDH2 and phosphorylated PI3K and AKT were markedly lower in the DM group than in the control group, which were reversed in the orientin-treated group (Figure 3B-C).

**Figure 3:** – Effects of orientin on the H19/miR-103-3p/ALDH2/PI3K/AKT axis in DM mice. (A) RT-qPCR data showing H19, miR-103-3p, and ALDH2 expressions in mouse hearts. One-way ANOVA. (B-C) Western blot showing levels of ALDH2 and phosphorylated PI3K and AKT in mouse hearts. One-way ANOVA. N=8 per group. ***p<0.01 vs control group; ###p<0.001 vs DM group.

### Orientin reduces high glucose-triggered oxidative stress and apoptosis in HL-1 cells

CCK-8 results indicated that orientin at doses of 2.5- 20 μM did not influence HL-1 cell viability (Figure 4A). Orientin at doses of 5, 10, and 20 μM rescued HL cell viability suppressed by high glucose (Figure 4B), and 10 μM orientin showed the most significant effects. We used 10 μM orientin in subsequent experiments. As presented in Figure 4C-F, high glucose enhanced ROS and MDA levels and decreased SOD activities, which were reversed by orientin pretreatment. Orientin pretreatment restored protein levels of Bax, Bcl-2, cleaved caspase3, ALDH2, and phosphorylated PI3K and AKT in high glucose-treated HL-1 cardiomyocytes (Figure 4G-H). Additionally, reduced expressions of H19 and ALDH2 and increased expression of miR-103-3p in high glucose-treated HL-1 cells were reversed by orientin pretreatment (Figure 4I).

**Figure 4:** – Orientin reduces high glucose-triggered oxidative stress and apoptosis in HL-1 cells. (A-B) HL-1 cell viability after orientin or/and high glucose treatment measured by CCK-8. One-way ANOVA. (C-F) Effects of orientin on ROS and MDA levels and SOD activities in HL-1 cells. One-way ANOVA. (G-H) Western blot showing levels of Bax, Bcl-2, cleaved caspase3, ALDH2, and phosphorylated PI3K and AKT in HL-1 cardiomyocytes. One-way ANOVA. (I) RT-qPCR data showing H19, miR-103-3p, and ALDH2 expressions in HL-1 cardiomyocytes. One-way ANOVA. N=3 per group. ***p<0.01 vs control group; ###p<0.001 vs DM group.

### The H19/miR-103-3p/ALDH2 axis regulates oxidative stress and apoptosis in HL-1 cells

The binding sites where H19 or ALDH2 interacts with miR-103-3p are shown in Figure 5A. The H19/ALDH2 sequence or its mutant version was co-transfected into HL-1 cells with miR-103-3p mimic. Data indicated significant decreases in luciferase activities following co-transfections with H19-Wt or ALDH1-Wt and miR-103-3p mimic, whereas co-transfections with H19-Mut or ALDH1-Mut and miR-103-3p mimic did not alter luciferase activities (Figure 5B). We found that overexpressing H19 restored protein levels of Bax, Bcl-2, cleaved caspase3, ALDH2, and phosphorylated PI3K and AKT in high glucose-treated HL-1 cardiomyocytes, which were reversed by miR-103-3p overexpression or ALDH2 depletion (Figure 5C-D). Additionally, H19 overexpression inhibited high glucose-triggered ROS production in HL-1 cells, but miR-103-3p overexpression or ALDH2 depletion negated the effects of H19 overexpression (Figure 5E).

**Figure 5:** – The H19/miR-103-3p/ALDH2 axis regulates oxidative stress and apoptosis in HL-1 cells. (A) Diagram depicting the binding sites between H19/ALDH2 and miR-103-3p. (B) Luciferase reporter assay showing luciferase activities following co-transfections with H19-Wt/Mut or ALDH1-Wt/Mut and miR-103-3p mimic. Two-way ANOVA. ***p<0.01 vs. NC mimic group. (C-D) Western blot showing levels of Bax, Bcl-2, cleaved caspase3, ALDH2, and phosphorylated PI3K and AKT in HL-1 cardiomyocytes. One-way ANOVA. (E) ROS levels in HL-1 cardiomyocytes. One-way ANOVA. N=3 per group. ***p<0.01 vs control group; ###p<0.001 vs HG + Vector + NC mimic + si-NC group; &&&p<0.001 vs HG + H19 group.

### H19 depletion or PI3K inhibitor reverses the effects of orientin in HL-1 cells

For the rescue experiments, HL-1 cardiomyocytes under high glucose conditions were treated with orientin or/and with H19 depletion or PI3K inhibitor (LY294002). Results showed that the changes in the levels of Bax, Bcl-2, cleaved caspase3, ALDH2, and phosphorylated PI3K and AKT caused by orientin treatment were notably reversed by H19 knockdown or LY294002 treatment (Figure 6A-B). Furthermore, the H19 knockdown or LY294002 treatment counteracted the effects of orientin on ROS and MDA levels and SOD activities in high glucose-treated HL-1 cells (Figure 6C-E).

**Figure 6:** – H19 depletion or PI3K inhibitor reverses the effects of orientin in HL-1 cells. (A-B) Western blot showing levels of Bax, Bcl-2, cleaved caspase3, ALDH2, and phosphorylated PI3K and AKT in HL-1 cardiomyocytes. One-way ANOVA. (C-E) ROS and MDA levels and SOD activities in HL-1 cells. One-way ANOVA. N=3 per group. ***p<0.01 vs control group; ###p<0.001 vs HG group; &&&p<0.001 vs HG + Orientin group.

## Discussion

In this study, orientin treatment alleviated deteriorated cardiac function in mice with DCM and protected against diabetes-induced cardiac fibrosis, apoptosis, and oxidative stress. Mechanistically, orientin restored the changes in the levels of H19, miR-103-3p, and ALDH2, as well as the PI3K/AKT pathway caused by hyperglycemia. This investigation, for the first time, reveals that the H19/miR-103-3p/ALDH2/PI3K/AKT axis has a key role in the cardioprotective action of orientin in DCM.

Orientin has been proposed as a promising compound for the treatment of cardiovascular disease. Orientin can exert antioxidant and antiapoptotic effects in ox-LDL-treated vascular endothelial cells and might protect against atherosclerosis.^33^ Orientin decreases inflammatory response, fibrosis, and cardiomyocyte apoptosis in mice following myocardial infarction. Additionally, orientin mitigates apoptosis and oxidative stress in cardiomyocytes exposed to hypoxia.^25,26^ Macrovascular complications of diabetes, including DCM, occur through multiple hyperglycemia-induced mechanisms in which oxidative stress has a central role.^34^ Cardiac oxidative stress is associated with reduced cardiac performance and contractility and increased cardiac fibrosis and hypertrophy, resulting in severe cardiac dysfunction and deadly cardiac events.^35^ Persistent hyperglycemia-mediated oxidative stress can activate apoptotic signaling in the heart to induce cardiomyocyte death.^36,37^ In this study, orientin did not influence blood glucose levels in DM mice. Still, it significantly ameliorated diabetes-induced cardiac dysfunction in mice independent of the modulation of blood glucose levels, possibly through inhibiting cardiac oxidative stress and apoptosis. M-mode echocardiography confirmed that orientin improved the hyperglycemia-induced cardiac dysfunction. Morphological and staining analyses further demonstrated that orientin ameliorated diabetes-induced cardiac tissue damage. LDH, CK-MB, and cTnI are key biochemical markers that reflect the degree of cardiac injury.^38^ Here, orientin markedly decreased LDH, CK-MB, and cTnI levels in the serum of DM mice.

The lncRNA-miRNA-mRNA ceRNA network has been reportedly involved in the regulation of the pathological processes of DCM.^39-41^ H19, a lncRNA abundant in the cardiovascular system, acts as a ceRNA to play a role in diabetes-related complications.^42,43^ Additionally, H19 is emerging as a potentially important regulator of cardiac disease.^44^ As reported, H19 overexpression improves left ventricular dysfunction in DM by reducing cardiomyocyte apoptosis and fibrosis.^14^ Shengjie Tongyu decoction protects against diabetic cardiac injury through upregulation of H19.^45^ Although it has been reported that orientin confers cardioprotection at the protein level, whether orientin could modulate the ceRNA network has not been investigated. Here, we confirmed that orientin restored H19 expression and decreased miR-103-3p expression *in vivo* and *in vitro*. H19 could bind to miR-103-3p as a sponge. miR-103-3p belongs to the miR-103/107 family and has been reported to increase cardiomyocyte programmed necrosis in myocardial ischemic injury.^46^

Additionally, miR-103-3p promotes hypertrophy and ROS production in the heart failure model.^47^ The effects of miR-103-3p in DCM are unknown. Here, we demonstrated that miR-103-3p negated the inhibitory effects of H19 overexpression on apoptosis and oxidative stress in HL-1 cells under high glucose conditions. Additionally, H19 could upregulate ALDH2, a target of miR-103-3p, in high glucose-treated HL-1 cells. Yu et al. demonstrated that ALDH2 is a crucial mediator in the cardioprotection of remote ischemic postconditioning through the PI3K/AKT-dependent pathway.^48^ ALDH2 alleviates ischemic injury in DCM through activation of the PI3K/AKT pathway.^19^ We found that the effects of H19 on the levels of apoptosis- and ALDH2/PI3K/AKT-related protein under high glucose conditions were reversed by miR-103-3p overexpression or ALDH2 knockdown, suggesting that H19 may activate the ALDH2/PI3K/AKT pathway by competing with miR-103-3p. It is noteworthy that orientin could upregulate ALDH2 expression in cardiomyocytes with high glucose. The effects of orientin on cardiomyocyte apoptosis and oxidative stress were reversed by H19 siRNA or an inhibitor of PI3K, demonstrating the key role of the H19/miR-103-3p/ALDH2/PI3K/AKT axis in the cardioprotective action of orientin.

Despite these findings, several limitations warrant consideration. Firstly, our in vivo model employed streptozotocin-induced diabetic mice fed a high-fat diet. While this replicates key features of human DCM, it does not fully capture the complexity of type 2 diabetes pathophysiology, including gradual β-cell dysfunction. Secondly, the study focused on the H19/miR-103-3p/ALDH2/PI3K/AKT axis as the primary mechanistic pathway; other signaling cascades or noncoding RNAs may contribute to orientin’s cardioprotective effects. Thirdly, long-term safety and pharmacokinetics of orientin in diabetic models remain unexplored. Future studies using genetic diabetes models, multi-omics approaches, and chronic toxicity assessments would strengthen clinical translation.

## Conclusion

In conclusion, our study demonstrates that orientin ameliorates DCM by attenuating oxidative stress and apoptosis through the H19/miR-103-3p/ALDH2/PI3K/AKT signaling axis. This work not only reveals a novel molecular mechanism for orientin’s cardioprotective effects but also identifies H19 and ALDH2 as potential therapeutic targets for DCM. Given orientin’s natural origin, these findings support its translational promise as a complementary strategy against diabetes-induced cardiac complications.
